# Which endpoints should we use in evaluating the use of novel fluoropyrimidine regimens in colorectal cancer?

**DOI:** 10.1038/sj.bjc.6600341

**Published:** 2002-06-05

**Authors:** C J Twelves, J Cassidy

**Affiliations:** Cancer Research UK Department of Medical Oncology, Alexander Stone Building, Garscube Estate, Switchback Road, Bearsden, Glasgow G61 1BD, Scotland, UK; Department of Medicine and Therapeutics, Institute of Medical Sciences, Foresterhill, Aberdeen AB25 2ZD, Scotland, UK

**Keywords:** colorectal cancer, 5-fluoropyrimidine, capecitabine, chemotherapy, oral, intravenous infusions, UFT, tegafur

## Abstract

Although significant advances have been made in the treatment of advanced/metastatic colorectal cancer, 5-fluorouracil (5-FU) still forms the basis of chemotherapy. Recently, new 5-FU schedules and novel fluoropyrimidines have been developed, but there are no trials directly comparing these regimens. The current review describes the mechanisms of action, pre-clinical and phase I/II studies of two oral fluoropyrimidine therapies, capecitabine and uracil with tegafur plus leucovorin. It also compares the phase III studies of these agents with those of the two most popular infusional 5-FU-based regimens: de Gramont and German AIO (The Association of Medical Oncology (AIO) of the German Cancer Society). Both oral and infusional regimens demonstrated similar survival to the Mayo Clinic regimen, a standard treatment for colorectal cancer. Therefore, other endpoints must be examined to decide optimum therapy, including response rates, time to disease progression, tolerability and patients' convenience. All four new therapies demonstrated superior safety profiles compared with the Mayo Clinic regimen. However the uracil with tegafur plus leucovorin regimen was associated with severe diarrhoea and capecitabine with hand–foot syndrome. Patients will not sacrifice efficacy for the convenience of oral therapy alone, therefore the fact that capecitabine achieved superior response rates and equivalent time to disease progression compared with the Mayo Clinic regimen, while uracil with tegafur plus leucovorin produced lower response rates and significantly inferior time to disease progression, is highly relevant in choosing treatment.

*British Journal of Cancer* (2002) **86**, 1670–1676. doi:10.1038/sj.bjc.6600341 www.bjcancer.com

© 2002 Cancer Research UK

## 

Colorectal cancer is the third most common cancer in men and women, accounting for 783 000 new cases and 437 000 deaths worldwide in 1990 (Pisani *et al*, 1999; Parkin *et al*, 1999). About 40–50% of patients develop metastatic disease. The aims of any therapy in patients with advanced colorectal cancer are to control symptoms, maintain or improve quality of life and ultimately to prolong survival. Recent meta-analyses confirmed that chemotherapy prolongs time to disease progression (TTP) and survival in patients with advanced or metastatic colorectal cancer, compared with best supportive care or observation/no chemotherapy (Colorectal Cancer Collaborative Group, 2000; Jonker *et al*, 2000).

The fluoropyrimidine, 5-fluorouracil (5-FU), has formed the basis of chemotherapy for colorectal cancer for over 40 years. Numerous 5-FU-based schedules are used, and extensive efforts have been made to increase their activity, including biomodulation, modification of the dose or schedule and the use of analogues/prodrugs.

The most successful biomodulation of 5-FU has been with leucovorin (LV), a derivative of tetrahydrofolic acid, the reduced form of folic acid. The rationale is that in the presence of reduced folate, fluorodeoxyuridine monophosphate (a metabolite of 5-FU), covalently interacts with thymidylate synthase, which is the source for *de novo* synthesis of thymidine nucleotides, ultimately disrupting DNA synthesis. In a meta-analysis, the addition of LV to 5-FU significantly improved response rates in patients with advanced colorectal cancer (23% *vs* 11%; *P*<10^−7^), although there was no difference in overall survival (Advanced Colorectal Cancer Meta-Analysis Project, 1992).

With regards to optimising the 5-FU schedule, continuous or protracted infusion is more effective than bolus administration in terms of response rates and TTP. In a phase III trial comparing continuous intravenous 5-FU infusion (750 mg m^−2^ day^−1^, daily for 7 days, every 21 days) with bolus administration (500 mg m^−2^ day^−1^, daily for 5 days, every 28 days) in patients with metastatic colorectal cancer, the response rate was significantly higher in the infusion group than the bolus group (26 *vs* 13%; *P*<0.04), (Rougier *et al*, 1997). Again, no difference in overall survival was observed. The superiority of continuous infusion was confirmed by a meta-analysis in which it achieved a significantly higher response rate than bolus 5-FU (22 *vs* 14%; *P*=0.0002) (Meta-analysis Group In Cancer, 1998). However, the increase in overall survival (12.1 months *vs* 11.3 months; *P*=0.04) was small and probably not clinically meaningful.

Raltitrexed is a direct, specific inhibitor of thymidylate synthase. It is structurally distinct from the fluoropyrimidines and has the advantage of being administered as an intravenous bolus every 3 weeks. Objective response rates with raltitrexed are similar to those with 5-FU plus LV (Cunningham, 1998) but recent trials have demonstrated inferior efficacy (TTP/survival) or raised safety concerns. Most recently, other agents with novel molecular targets have shown clear evidence of activity in colorectal cancer. Irinotecan prolongs survival in colorectal cancer, both as first-line therapy when added to 5-FU plus LV (Douillard *et al*, 2000; Saltz *et al*, 2000) and in second-line therapy as a single agent when compared with best supportive care or infusional 5-FU (Cunningham *et al*, 1998; Rougier *et al*, 1998). Of note, this improvement in survival required the use of a new family of drugs directed to a novel target, whereas survival differences have been difficult to identify in trials of fluoropyrimidines. Similarly, oxaliplatin substantially increases response rates when added to infusional 5-FU plus LV, although improved overall survival has not yet been demonstrated (de Gramont *et al*, 2000; Giacchetti *et al*, 2000).

The Mayo Clinic regimen is a commonly used 5-FU treatment, which has confirmed efficacy (Poon *et al*, 1991). It comprises bolus LV, 20 mg m^−2^ followed by bolus 5-FU, 425 mg m^−2^, both administered daily for 5 days, every 4 weeks. The Mayo Clinic regimen in various forms has been used as the control arm in numerous colorectal cancer clinical trials (de Gramont *et al*, 1997; Aranda *et al*, 1998; Cunningham, 1998; Carmichael *et al*, 1999; Pazdur *et al*, 1999; Young *et al*, 1999; Buechele *et al*, 2000; Maughan *et al*, 2000; Saltz *et al*, 2000, 2002; Schmoll *et al*, 2000; Wang *et al*, 2000; Hoff *et al*, 2001; Van Cutsem *et al*, 2001; Twelves, 2002).

This review describes four phase III studies in detail, concentrating on the significance of different endpoints in colorectal cancer. Two of these studies investigated the oral therapies, capecitabine (Xeloda®), and a combination of UFT (uracil and ftorafur, also known as tegafur (Orzel™, Uftoral™) plus LV. The mechanisms of action, and pre-clinical and phase I/II studies of capecitabine and UFT plus LV are also described. The other studies investigated two of the most popular infusional 5-FU-based regimens, the de Gramont and the German AIO regimens.

Investigation of endpoints across these different trials is important because these new oral fluoropyrimidines and infusional regimens have not been directly compared in clinical trials, and such trials are unlikely in the future. Although it is not necessarily the most widely used regimen, the Mayo Clinic is useful as a common comparator across clinical trials.

## ORAL REGIMENS

### Capecitabine

Capecitabine has recently been approved in Europe and the USA for use in advanced colorectal cancer. It is a novel fluoropyrimidine carbamate, rationally designed to be taken orally and through a three step process generate 5-FU preferentially in tumour tissue. Thus, capecitabine mimics protracted 5-FU infusion but with a more convenient mode of administration. It also potentially reduces systemic exposure to 5-FU, thereby improving the therapeutic index (Di Costanzo *et al*, 2000). Pre-clinical work showed that capecitabine had superior anti-tumour activity in various human cancer xenograft models compared with UFT or 5-FU (Ishikawa *et al*, 1998b), as well as anti-tumour activity in 5-FU-sensitive and 5-FU-resistant tumours (Cao *et al*, 1997).

Capecitabine is not itself cytotoxic but is first converted to 5′-deoxy-5-fluorocytidine (5′-DFCR) by carboxylesterase, located primarily in the liver. Next, 5′-DFCR is converted to 5′-deoxy-5-fluorouridine (5′-DFUR) by cytidine deaminase, present mainly in the liver and tumour tissue. Finally, 5′-DFUR is converted to 5-FU by thymidine phosphorylase, which has significantly higher activity in tumour than normal tissues (Ishikawa *et al*, 1998a). Preferential activation of capecitabine to 5-FU in patients was demonstrated in a recent study, in which exposure to 5-FU in primary colorectal tumours was on average 3.2 times higher than in adjacent healthy tissue (*P*=0.002), and 21.4 times higher than in plasma (Schüller *et al*, 2000).

In a phase I study using an intermittent schedule (2 weeks treatment followed by a 1-week rest period), the dose-limiting toxicities (DLTs) were diarrhoea with hypotension, abdominal pain and leucopenia (Mackean *et al*, 1998). Other phase I studies evaluated a continuous schedule (Budman *et al*, 1998) and the combination of capecitabine with oral LV (Cassidy *et al*, 1998). A randomised phase II study in colorectal cancer patients comparing these three schedules reported response rates of 24, 21 and 23%, respectively; all three schedules were generally well tolerated (Van Cutsem *et al*, 2000). Based on considerations of toxicity, dose-intensity, response rate and TTP, the intermittent capecitabine monotherapy regimen (2-weeks treatment, 1-week rest) was selected for subsequent phase III studies, with a recommended starting dose of 1250 mg m^−2^ twice daily.

### UFT plus LV

UFT is an orally administered combination of tegafur and uracil in a fixed 1 : 4 molar ratio that has been available in Japan since 1984. Tegafur is a 5-FU prodrug that is converted to 5-FU by hepatic microsomal cytochrome P450 enzymes, or by ubiquitous cytosolic enzymes. The rationale for the addition of uracil is that it competes with 5-FU as a substrate for dihydropyrimidine dehydrogenase, the rate-limiting enzyme responsible for 5-FU catabolism, thus preventing the typical rapid breakdown of 5-FU. In high doses, tegafur is associated with neurological adverse effects, including depression, headache and dizziness, in which the metabolite alpha–fluoro–beta–alanine appears to play a crucial role. Uracil greatly reduces the dose of tegafur used, thus reducing the concentration of this metabolite. In animals, the co-administration of uracil and tegafur reduced tegafur neurotoxicity (Yamamoto *et al*, 1984). The rationale for the addition of LV to UFT follows that of adding LV to 5-FU. Evidence for the benefit of adding LV to UFT comes from a study in which LV significantly enhanced the growth-suppressive ability of UFT against human colon and mammary tumour xenografts (Okabe *et al*, 1997).

The MTD of UFT plus LV was determined in several phase I studies using UFT administered in divided doses for 14 days every 4 weeks or for 28 days every 5–6 weeks; LV doses varied 10-fold (Hoff, 2000). The DLTs consisted of diarrhoea, vomiting, fatigue, stomatitis, abdominal pain, leucopenia and abnormal liver function tests. In phase II trials, UFT plus LV achieved response rates between 0 and 42%. However, these trials suggest a narrow dose–toxicity relationship, given that lowering the UFT dose from 350 to 300 mg m^−2^ day^−1^ markedly reduced the incidence of grade 3 diarrhoea from 71 to 11% (Hoff, 2000; Pazdur *et al*, 1994). Based on these results, UFT 300 mg day^−1^, with LV 75–90 mg day^−1^, was given for 28 days and repeated every 5 weeks in phase III colorectal cancer studies.

## ORAL AND INFUSIONAL REGIMENS *VS* MAYO CLINIC REGIMEN – PHASE III DATA

Table 1

**Table 1:**
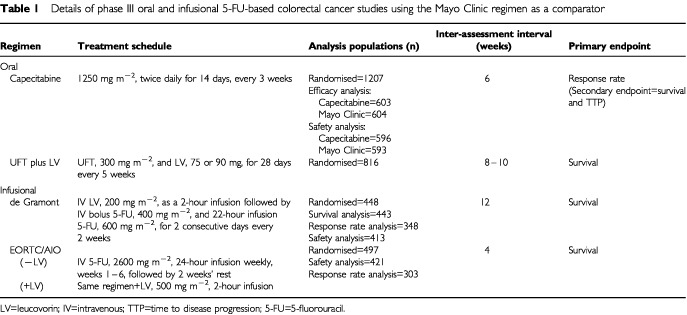
Details of phase III oral and infusional 5-FU-based colorectal cancer studies using the Mayo Clinic regimen as a comparator

shows the four phase III trials of first-line treatment for colorectal cancer to be discussed, using the Mayo Clinic regimen as the comparator. The novel treatments investigated were oral capecitabine (Twelves, 2002), UFT plus LV (Pazdur *et al*, 1999), and two infusional 5-FU-based regimens (de Gramont *et al*, 1997; Schmoll *et al*, 2000). The capecitabine data comprises pooled results from two phase III trials, with identical protocols and conduct. One study was conducted in Europe, Australia and Asia (Van Cutsem *et al*, 2001), and the other in the USA, Brazil and Mexico (Hoff *et al*, 2001). The UFT plus LV trial was performed in Europe, the USA and Canada, while the de Gramont regimen trial was performed in France. The EORTC/AIO study was a 3-arm trial, with infusional 5-FU given with or without LV, in which the Mayo Clinic regimen was administered every 4 weeks for two cycles and then every 5 weeks. Additional information on these trials is cited elsewhere (Benner, 1999; Cassidy, 2000; Cunningham and James, 2001).

Although the studies used the same comparator regimen, differences are evident. These include inter-assessment intervals, which is pertinent as longer periods can result in cruder estimation of some endpoints such as TTP (Cassidy, 2000). Furthermore, the analysis population was substantially smaller than the all randomised population in the de Gramont trial, and only patients with measurable disease were included in the response rate analysis in the EORTC/AIO study. The trials with the oral compounds were for registration purposes, so use of the all randomised population for efficacy parameters was mandatory and safety was monitored intensively.

### Efficacy

None of the four trials achieved a significant survival advantage over the Mayo Clinic regimen (Table 2

**Table 2:**
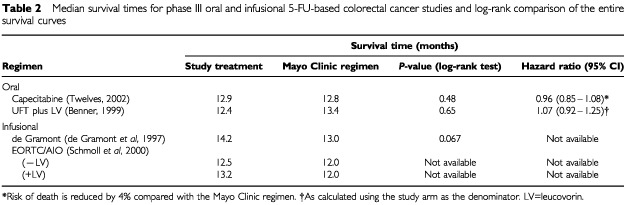
Median survival times for phase III oral and infusional 5-FU-based colorectal cancer studies and log-rank comparison of the entire survival curves

). Only the capecitabine studies used response rate as a primary endpoint, these trials being designed and statistically powered to demonstrate equivalent rather than superior survival. Capecitabine achieved significantly higher response rates than the Mayo Clinic regimen (*P*<0.0002), confirmed by independent review committee (IRC) assessment (Table 3

**Table 3:**
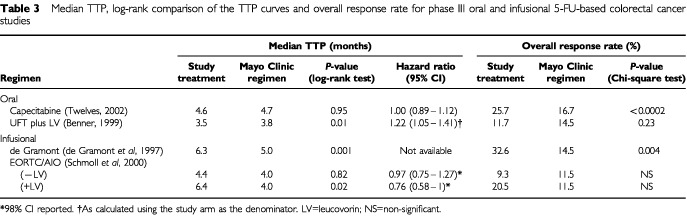
Median TTP, log-rank comparison of the TTP curves and overall response rate for phase III oral and infusional 5-FU-based colorectal cancer studies

). Moreover, capecitabine showed a consistent and significantly higher (*P*<0.05) response rate in all subpopulations analysed (prior adjuvant therapy, predominant site of metastases, single/multiple sites and Karnofsky Performance Status) (Twelves, 2002). Capecitabine produced an equivalent median TTP to the Mayo Clinic regimen (Table 3).

**Figure 1:**
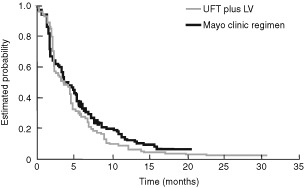
Time to disease progression Kaplan–Meier curve for the UFT plus LV *vs* Mayo Clinic regimen phase III trial (Benner, 1999).

In contrast to the results obtained with capecitabine, UFT plus LV produced a lower response rate than the Mayo regimen, although this difference was not statistically significant. TTP was, however, significantly inferior with UFT plus LV compared to the Mayo Clinic regimen and recalculating published data (Benner, 1999) using the study arm as the denominator in line with the capecitabine study, the hazard ratio was 1.22 (*P*=0.01). This equates to a 22% increased risk of disease progression, with UFT plus LV compared with the Mayo Clinic regimen (Figure 1

). A second phase III trial involving 380 patients, compared UFT/LV with a non-standard Mayo Clinic regimen (of 5-FU/LV given every 5 weeks, rather than 4 weekly). In this trial UFT/LV produced no significant difference in survival, response rates or median TTP (Carmichael *et al*, 1999). However, as highlighted by the US Food and Drug Administration (FDA), the control arm in this study had a planned 25% lower dose intensity than the standard Mayo Clinic regimen. Although the difference in delivered dose intensity was less, this may explain the differences in results between the two UFT plus LV phase III studies (Benner, 1999). Moreover, there are insufficient data on the efficacy of this non-standard Mayo Clinic regimen for it to be a valid comparator.

The de Gramont regimen achieved a statistically significant improvement in response rates. However, the response rate analysis was based on only three quarters of the randomised population. A large difference was observed in response rates between the EORTC/AIO (20.5%) and Mayo Clinic regimens (11.5%), but this did not reach statistical significance, most likely because relatively few patients had measurable disease. Both the de Gramont and EORTC/AIO plus LV regimens produced modest, but statistically significant, improvements in median TTP, although estimates in the de Gramont study are cruder due to the longer inter-assessment intervals.

In summary, the infusional regimens produced significant improvements in TTP, whereas UFT plus LV produced a significantly inferior TTP; oral capecitabine and the de Gramont regimen resulted in significant improvements in response rates.

### Safety profiles

Since therapy in this setting is generally given with palliative intent, it is important to consider toxicity carefully. In terms of overall incidence, capecitabine caused significantly less diarrhoea, nausea, stomatitis and alopecia compared with the Mayo Clinic regimen (Hoff *et al*, 2001; Van Cutsem *et al*, 2001) and UFT plus LV significantly less diarrhoea, neutropenia, nausea/vomiting and stomatitis/mucositis (Pazdur *et al*, 1999).

Looking at grade 3 or 4 toxicities, all four regimens were associated with less severe neutropenia/leucopenia (all differences statistically significant, apart from EORTC/AIO) and a lower incidence of severe stomatitis/mucositis than the Mayo Clinic regimen. With the exception of capecitabine, there was a trend towards a higher incidence of grade 3 or 4 nausea and vomiting with the new regimens in all trials. Grade 3/4 diarrhoea was significantly less frequent with the de Gramont than the Mayo Clinic regimen, while the EORTC/AIO plus LV regimen was associated with more diarrhoea than the EORTC/AIO regimen without LV and the Mayo Clinic regimen. No significant difference in the incidence of grade 3 or 4 diarrhoea was observed with capecitabine compared with the Mayo Clinic regimen. However, analysis of the published data by Fisher's Exact test (Benner, 1999; Pazdur *et al*, 1999) showed a trend for increased incidence of grade 3 or 4 diarrhoea with UFT plus LV (*P*=0.057), based on a safety population of 406 patients for UFT plus LV and 396 for the Mayo Clinic regimen (Cunningham and James, 2001). This high incidence of grade 3 or 4 diarrhoea (21%) has also been observed in a phase III adjuvant trial (26%) (Smith *et al*, 1999).

Hand–foot syndrome, a cutaneous condition affecting palms and soles, is one of the more common adverse effects of capecitabine, but is usually mild or moderate in intensity. In the capecitabine trial, only two out of the 596 patients included in the safety analysis were hospitalised because of this adverse effect (one patient required an 8-h stay and one needed overnight observation). Hand–foot syndrome was effectively managed by use of emollients, treatment interruption or, if necessary, dose reduction which prevented recurrence of grade 2/3 hand–foot syndrome in all but 45 out of 171 patients (Cassidy and Twelves, 2000). Efficacy was maintained in those patients who required dose modification, as indicated by a Cox regression analysis of TTP in patients with and without dose reduction, in which the hazard ratio was 0.97 (*P*=0.78).

## ORAL AND INFUSIONAL REGIMENS – TREATMENT CHOICE

It is clear that neither the oral nor infusional fluoropyrimidine regimens discussed offer any significant benefit in overall survival compared with the Mayo Clinic regimen. Likewise, although continuous infusion of 5-FU achieves higher response rates than the Mayo Clinic regimen, this does not impact on survival (Aranda *et al*, 1998; Rougier *et al*, 1998). Therefore, other factors must be taken into consideration when choosing appropriate chemotherapy, including TTP, response rates and tolerability.

Infusional regimens are time-consuming, inconvenient and uncomfortable for the patient, and often require regular hospital visits. They are also frequently associated with venous access-related complications, such as infections, sepsis, thrombosis and blockage. In subsets of patients with in-dwelling central venous catheters, more than 60% develop upper extremity deep vein thrombosis (Prandoni and Bernardi, 1999) and infection rates of 10–30% have been reported (Clark and Raffin, 1990). The administration schedule of raltitrexed as an intravenous infusion over 15 min, repeated every 3 weeks, offers increased convenience for the patient. Raltitrexed was compared with 5-FU in four large, randomised trials. In two of these trials the Mayo regimen was the comparator, so these studies can be placed alongside those discussed above (Cunningham, 1998). In both trials the objective response rates were very similar to those with the Mayo Clinic regimen. In one trial, survival also was identical. However, in the other trial, median survival was significantly worse with raltitrexed than with 5-FU plus LV (9.7 and 12.7 months, respectively; *P*=0.01). There was also a marked difference in duration of chemotherapy, with patients remaining on treatment for substantially longer in the 5-FU plus LV arm. Although in some studies raltitrexed was better tolerated than bolus 5-FU plus LV, in another it was associated with an excess of treatment-related deaths in comparison with infusional 5-FU (Maughan *et al*, 2000). The development of raltitrexed in the adjuvant setting was abandoned because of these concerns.

Oral therapy overcomes the delivery problems associated with infusional regimens and a recent questionnaire study showed that patients have a strong preference for oral rather than intravenous treatment (Liu *et al*, 1997). Of 103 assessable patients with incurable cancer, 89% expressed a preference for oral rather than intravenous chemotherapy. The main reasons for preferring oral treatment were convenience (57%), problems with intravenous lines (55%) and control over the environment in which they received chemotherapy (33%). However, more than two thirds of patients did not want to sacrifice response rate (70%) or response duration (74%) for the convenience of oral treatment. Patient preference for oral administration was confirmed by a prospective clinical study, in which 84% of patients preferred oral UFT plus LV to intravenous 5-FU plus LV (Borner *et al*, 2002).

Given that patients are reluctant to sacrifice efficacy for convenience, the fact that capecitabine produced superior response rates and equivalent TTP compared with the Mayo Clinic regimen, while UFT plus LV produced lower response rates and inferior TTP, is relevant when considering treatment options. Patients' reluctance to sacrifice efficacy is also relevant when considering the use of raltitrexed. Although raltitrexed may have a place in the treatment of some patients who cannot tolerate 5-FU (Taylor, 2000), the results of clinical trials question its efficacy compared to commonly used 5-FU/LV regimens. Unlike intravenous regimens, oral agents are not associated with risk of local toxicity related to their administration or placement of an in-dwelling central venous catheter. For the patient, oral chemotherapy substantially reduces the amount of time spent at the hospital for treatment and is clearly more convenient (Twelves *et al*, 2001). However, another factor to be considered when comparing oral therapies is the timing of treatment. UFT plus LV should be given every 8 h, at least 1 h before or after meals, creating practical difficulties that may impact upon quality of life. By contrast, no clinically relevant differences in the pharmacokinetics of capecitabine and its metabolites were observed in patients taking capecitabine under fasting conditions or after a standard meal (Reigner *et al*, 1998). It is recommended that capecitabine be administered twice daily within 30 min after breakfast and an evening meal with water to mimic the clinical trials, but this should cause little if any disruption to patients' lifestyles.

The considerable advantages associated with oral agents mean that they will soon find a place in routine practice (Young and Rea, 2000). With capecitabine these advantages also extended to substantial savings in medical resource use (Twelves *et al*, 2001).

## SUMMARY

The ultimate aim in the treatment of any patient with colorectal cancer is to increase their survival, but neither the oral nor infusional fluoropyrimidine therapies discussed achieved this goal compared with the standard Mayo Clinic regimen. Furthermore, patients and physicians face practical problems when choosing between the new fluoropyrimidines, as many clinicians use one of the other infusional treatments rather than the Mayo Clinic regimen. In the absence of direct, comparative trials between these regimens, clinicians have no choice but to make the best indirect comparisons possible, using the Mayo Clinic regimen as the common link.

Both oral capecitabine and the infusional de Gramont regimens significantly improved response rates compared with the Mayo Clinic regimen. Moreover, the de Gramont and EORTC/AIO plus LV infusional regimens significantly, but modestly, improved TTP. In contrast, UFT plus LV resulted in a 22% increased risk of disease progression. All four therapies demonstrated superior safety profiles compared with the Mayo Clinic regimen although differences in the type and severity of adverse effects were observed. In terms of patients' convenience, oral therapy is preferable to infusional regimens, but patients will not sacrifice efficacy for convenience. This would favour the choice of capecitabine over UFT plus LV.

The treatment of colorectal cancer is evolving rapidly, with one focus the choice between combination and sequential chemotherapy. Efficacy can be enhanced by combining 5-FU/LV with either irinotecan (Cunningham *et al*, 1998; Rougier *et al*, 1998; Douillard *et al*, 2000; Saltz *et al*, 2000) or oxaliplatin (de Gramont *et al*, 2000; Giacchetti *et al*, 2000). A logical step forward would be to use an oral fluoropyrimidine, in combination with oxaliplatin or irinotecan, or with radiotherapy in patients with rectal cancer. Already, several phase I/II studies have been undertaken combining UFT or capecitabine with oxaliplatin or irinotecan. In the setting of first-line combination therapy, where increased survival has already been shown, the evidence that capecitabine is the more active of the oral fluoropyrimidines suggests it is an especially attractive partner for oxaliplatin and irinotecan. The definitive answer as to which is the optimal combination will depend on large-scale, comprehensive studies, the results of which are eagerly awaited.
